# The Green Lady Hostel

**Published:** 1910-10-08

**Authors:** Lily H. Montagu

**Affiliations:** 12 Kensington Palace Gardens, W.


### Editor's Letter-Box.

### THE GREEN LADY HOSTEL.

*To the Editor of* The Hospital.

Dear Sir,—I venture to hope that you will kindly allow me to appeal to your many readers through your valuable paper, for they, having just returned from their own holidays, may be interested to hear about our Working People's Hostel at Littlehampton. This hostel is managed by a council with a working executive, and is in the hands of trustees. The house has a beautiful garden and is situated near the sea. It can accommodate at one time about fifty-two girls, who thus have an opportunity of sharing with their club leaders a chance of real recreation during at least one week out of their busy working lives. The hostel is run on an entirely unsectarian basis. Each boarder pays 10s. a week towards her maintenance, and while the hostel is full the payments make it entirely self-supporting; but, unfortunately, we have to provide for the winter months, and also for the salary of the matron who looks after the housekeeping matters and renders her guests thoroughly comfortable.

It is for this reason that we appeal for the sum of £500, which would place our work on a permanently satisfactory basis. During this summer some six hundred people have enjoyed the hospitality of the Green Lady Hostel, and have come back with renewed hope and vigour to take up the burdens of their strenuous lives, feeling that these burdens are lightened by the memory of the happy time they have spent in really delightful surroundings.

I shall be glad to supply any further particulars or acknowledge with grateful thanks any donations which your readers may be good enough to forward to me.

I am,

Yours faithfully,

Lily H. Montagu.

12 Kensington Palace Gardens, W.

